# Polarisation of a T-helper cell immune response by activation of dendritic cells with CpG-containing oligonucleotides: a potential therapeutic regime for bladder cancer immunotherapy

**DOI:** 10.1038/sj.bjc.6601474

**Published:** 2003-12-09

**Authors:** H Atkins, B R Davies, J A Kirby, J D Kelly

**Affiliations:** 1 Northern Institute for Cancer Research, School of Surgical and Reproductive Sciences, Faculty of Medical Sciences, University of Newcastle, Framlington Place, Newcastle-Upon-Tyne NE2 4HH, UK

**Keywords:** bladder cancer, BCG immunotherapy, CpG-oligonucleotides, toll-like receptor

## Abstract

Intravesical bacillus Calmette–Guerin (BCG) is a treatment for transitional cell carcinoma (TCC) and carcinoma *in situ* (*cis*) of the urinary bladder, but some patients remain refractory. The mechanism of cancer clearance is not known, but T cells are thought to play a contributory role. Tissue dendritic cells (DCs) are known to initiate antigen-specific immune responses following activation of receptors, which recognise molecular patterns on the surface of microorganisms. A family of these receptors, the toll-like receptors (TLRs), are also crucial for activating DC to produce cytokines that polarise the T-cell response towards a T helper (Th)1 or Th2 phenotype. This study compared the potential of intact BCG to activate DC with that of the defined TLR4 ligand lipopolysaccharide (LPS) and the TLR9 ligand CpG-oligonucleotide. It was found that all three stimuli efficiently activated normal DC, but cells expressing a mutant TLR4 responded poorly to stimulation with LPS. Importantly, stimulation with BCG induced both IL-12 and IL-10, suggesting subsequent development of a poorly focused T-cell immune response containing both Th1 and Th2 immune function. By contrast, LPS- and CpG-oligonucleotides induced only IL-12, indicating the potential to produce a Th1 response, which is likely to clear cancer most efficiently. Given the toxicity of LPS, our data suggest that CpG-oligonucleotides may be beneficial for intravesical therapy of bladder cancer.

Bladder cancer is a major health problem, with approximately 12 000 new cases diagnosed and 5000 deaths each year in England and Wales (Cancer Research UK, 2001). Intravesical *Mycobacterium bovis* bacillus Calmette–Guerin (BCG) has been used as an ablative treatment for carcinoma *in situ* (*cis*) of the bladder since 1976 (Morales *et al*, 1976) and is now accepted as a bladder-sparing treatment for *cis* with an early complete response of 70%; 40% of patients remain disease free after 5 years (Griffiths *et al*, 2002). Intravesical BCG is also widely used as an adjuvant to resection for the prophylaxis of recurrent superficial transitional cell carcinoma (TCC), and may delay progression to invasive disease (Sylvester *et al*, 2002). Although BCG has also been demonstrated to be superior to chemotherapy in a prospective randomised trial (Lamm *et al*, 1991; Malmstrom, 2000), 30% of TCC, including high-stage and muscle-invasive disease fail to respond. Furthermore, the administration of BCG results in local and systemic side effects including cystitis, polyarthritis and, rarely, death. Clearly there is scope to improve this therapy.

Recent work has provided strong evidence that the antitumour mechanism of BCG therapy is mediated through induction of a cascade of immunological events and the promotion of acute inflammation in the urothelium. This immune stimulation may be mediated through dendritic cells (DCs) that act as potent antigen-presenting cells and are capable of initiating cancer-directed immune responses (Jefford *et al*, 2001; Gabrilovich, 2002).

Dendritic cell activation in response to BCG is mediated by a family of innate immune receptors called toll-like receptors (TLRs), which recognise pathogen-associated microbial products (Medzhitov, 2001). Toll-like receptors signal through the intracellular MAPK and NF*κ*B pathway cascades to induce inflammatory cytokine secretion, induction of DC antigen presentation state and T-cell stimulation. Hence, ligation and signalling through TLRs can shape the developing immune response.

Effective BCG therapy requires induction of a T-cell-mediated immune response (Ratliff *et al*, 1987; Ratliff, 1992) and the activation of a natural killer (NK) cell-like population with an apparent ability to distinguish between normal and cancer cells (Bohle *et al*, 1993; Brandau *et al*, 2001). Significantly, both T-cell- and NK-cell-deficient mice respond poorly to BCG therapy (Brandau *et al*, 2001). Following BCG instillation, it has been shown that there is an accumulation of immunocompetent cells into the bladder wall including activated T helper (Th) lymphocytes, NK cells and DCs; adhesion molecules (ICAMs) and costimulatory B7 antigens are also upregulated (Patard *et al*, 1996; Saint *et al*, 2001). These activated effector cells kill target cells by production of nonspecific soluble factors and by direct cell-to-cell contact.

Many cytokines are detectable in the urine following BCG therapy, including IL-1, IL-2, IL-6, IL-7, IL-10, IL-12, interferon-inducible protein 10, TNF*α*, IFN*γ* and a number of chemokines (Jackson *et al*, 1995; Patard *et al*, 1998; Taniguchi *et al*, 1999). This range of cytokines indicates the activity of mutually antagonistic Th1-type and Th2-type immune processes with no evidence of polarisation of the response to a tissue-destructive and potentially more beneficial Th1 (or delayed type hypersensitivity; DTH) phenotype.

Importantly, animals with deletion of IL-12 or IFN*γ* are resistant to BCG immunotherapy and die rapidly from experimental bladder cancer (Riemensberger *et al*, 2002), while supplementing BCG with these characteristic Th1 cytokines can enhance cancer clearance (O'Donnell *et al*, 1999). By contrast, the production of immunosuppressive IL-10 during BCG therapy reduces inflammation and the anticancer response (Nadler *et al*, 2003). On this basis, we postulate that the immune response elicited by BCG is suboptimal and potentially antagonistic, and might limit the survival of some patients with bladder cancer. Furthermore, poor polarisation of the phenotype of the Th response may allow tumour-immune escape, suggesting a possible mechanism for BCG treatment failure.

In this report, we demonstrate that BCG stimulates cultured murine DCs to produce cytokines that potentially elicit a suboptimal, mixed Th1 and Th2 immune response. Moreover, in contrast to lipopolysaccharide (LPS), BCG is still able to stimulate the production of both Th1- and Th2-promoting cytokines by cultured DCs from TLR4-mutant mice. Significantly, we also show that stimulation of DCs with unmethylated CpG-motif containing oligonucleotides, a known ligand for TLR9, results in the production of IL-12 but not IL-10, suggesting that these agents polarise the immune response towards an antitumour Th1 phenotype. We suggest that CpG-oligonucleotides could have potential as immunotherapy agents in patients with bladder cancer.

## MATERIALS AND METHODS

### Animals

Female C3H/HeSn (TLR4 wild type, LPS responsive) and C3H/HeJ (TLR4 mutant, LPS resistant) mice (8–10 weeks old) were purchased from Charles River UK (Kent, UK). Female CD1 mice (8–10 weeks old) were purchased from Harlan UK (Oxon, UK). All mice were used in accordance with UK Home Office License (PPL60/2610).

### Generation of DCs from murine bone marrow

Femurs and tibiae were removed from mice following killing. Bones were sterilised by brief washing in 70% (v v^−1^) ethanol and the marrow flushed out using complete medium (RPMI-1640 supplemented with 10% (w v^−1^) foetal bovine serum, 100U ml^−1^ penicillin and 100 *μ*g ml^−1^ streptomycin and 2 mM L-glutamine (Sigma, Dorset, UK) using a 21-gauge needle attached to a 1 ml syringe. Following centrifugation, red blood cells were lysed by washing in 0.15 M ammonium chloride, 0.01 mM EDTA, 10 mM sodium bicarbonate. After further centrifugation, cell clusters were disassociated by vigorous syringing. Approximately 1–1.5 × 10^7^ leucocytes were obtained from each C3H mouse. Cells were resuspended in complete medium and seeded into either 24- or six-well tissue culture plates at a seeding density of 2 × 10^5^ or 2 × 10^6^ cells well^−1^, respectively. These cultures were supplemented with 10 ng ml^−1^ recombinant murine GM-CSF with or without 10ng ml^−1^ recombinant murine IL-4 (PeproTech EC, London, UK) and incubated at 37°C in a humidified atmosphere containing 5% (v v^−1^) CO_2_ for 7 days. On the third and fifth days of culture, 0.5 or 1 ml of medium was removed from each well of a 24- or six-well plate, respectively, and replaced with medium containing fresh cytokines. Fresh medium and cytokines were also added prior to all stimulations.

### Cell surface marker analysis by flow cytometry

Dendritic cells were examined by flow cytometry to verify their phenotype before and following activating stimuli. In all, 2 × 10^5^ cells were harvested and resuspended in PBS containing 5% (v v^−1^) FCS and examined for CD83 and CD86 expression by staining with monoclonal antibodies to CD83 (fluorescein isothiocyanate (FITC) conjugate, clone Michel17, rat IgG1, Biocarta, UK) and CD86 (B7-2, phycoerythrin (PE)-conjugated clone RMMP-2, rat IgG2a, Caltag, UK). Unstained cells were included as negative controls in each labelling experiment. Stained cells were analysed by flow cytometry (FACSort; Becton Dickenson, Cowley, UK). Data analysis was performed using FACs express software (DeNovo Software, Ontario, Canada).

### Endocytosis assay

Dendritic cell cultures were incubated with 1 mg ml^−1^ of FITC-labelled dextran (MW 70000, Sigma) for 60 min at either 37 or 4°C. Dendritic cells were harvested and washed three times in PBS containing 1% (v v^−1^) FCS and 0.01% (w v^−1^) sodium azide (NaN_3_). FITC-dextran uptake was analysed by FACs.

### Stimulation of DCs with BCG, LPS and CpG-oligonucleotides

Dendritic cell cultures (7 days old) were provided with fresh RPMI-1640 culture medium containing fresh cytokines (10 ng ml^−1^ GM-CSF, 10ng ml^−1^ IL-4); experimental wells were further supplemented with TLR ligands. These activating ligands were 50 or 100 ng ml^−1^ LPS (Sigma), 1.25 × 10^5^ colony forming units (CFU) ml^−1^ BCG (ImmuCyst™, Connaught strain, Pasteur Merieux Connaught, Canada), 2 or 5 *μ*M phosphorothioate-stabilised CpG-rich adjuvant oligonucleotide (ODN) (containing CG motif, ATA ATC GAC GTT CAA GCA AG; TAG, Newcastle, UK) or 2 *μ*M phosphorothioate stabilised non-CpG-rich control-ODN (reversed CG motif, ATA ATG CAG CTT CAA GCA AG; TAG Newcastle). The use of CpG-rich and non-CpG-rich ODN sequences has been described previously (Chu *et al*, 1997). All DC cultures were stimulated at 37°C for 72 h.

### Measurement of DC cytokine profiles by ELISA

Following stimulation with activating ligands, DC-conditioned media were collected and frozen at −20°C prior to analysis. The presence of IL-12 and IL-10 was measured by ELISA (murine IL-12 ELISA development kit, PeproTech EC, IL-10 eBioscience, San Diego, CA, USA). The results are presented as means±s.e. from duplicate analysis.

### Isolation of splenic lymphocytes

Mononuclear cells were isolated from allogenic mouse spleens using gradient separation and plastic-adherence depletion. Briefly, the spleens from CD1 mice were removed and passed through a 70 *μ*M nylon cell strainer (BD Falcon) followed by washing in complete medium. Cells were diluted with complete medium and underlain with 6 ml Histopaque™-1083 (Sigma Diagnostics, USA). Cells were centrifuged at 400 **g** for 25 min with zero break speed. Mononuclear cells were recovered from the interphase and monocytes depleted by plastic adherence for 2 h. The enriched lymphocyte population was washed in complete medium and used immediately in mixed lymphocyte reaction (MLR) assays.

### Measurement of DC-induced T-cell proliferation following BCG activation by the MLR assay

The functional maturity of stimulated DCs was assessed by investigation of their capacity to stimulate allogenic T-cell proliferation within an MLR. Briefly, 7-day-old cultures of both C3H/HeSn and C3H/HeJ DCs were stimulated with 6.25 × 10^4^ or 1.25 × 10^5^ CFU ml^−1^ BCG. After 72 h stimulation, DCs were irradiated with 25 Gray (Gy) *γ*-irradiation to prevent their proliferation during the course of the MLR. This dose of irradiation has previously been shown to be sufficient to prevent DC proliferation (Pettit *et al*, 2002).

Dendritic cells were added to the wells of a round profile 96-well plate in halving dilutions to give stimulator cell numbers ranging from 1 × 10^5^ to 1.25 × 10^3^ cells well^−1^. Responder T cells were added at 1 × 10^5^ cells well^−1^ such that the stimulator : responder ratio ranged from 1 : 1 to 0.125 : 1. T cell-only control cultures were also included and repeatedly showed low levels of proliferation. All cells were plated into complete medium in a total volume of 200 *μ*l. The proliferation of responder cells in each microculture was determined after 5 days following incubation with 1 *μ*Ci well^−1^ [3H]thymidine for 6 h. Radiolabelled DNA was harvested and [3H]thymidine incorporation measured using a *β*-scintillation counter. Assays were conducted in triplicate for each stimulator : responder ratio.

### Statistical analysis

All determinations were performed in triplicate and each result expressed as the mean±s.e. Statistical significance was determined by unpaired *t*-tests. A *P*-value of 0.05 or less was considered to be significant.

## RESULTS

### Characterisation of DCs from mouse bone marrow progenitors

**Figure 1:**
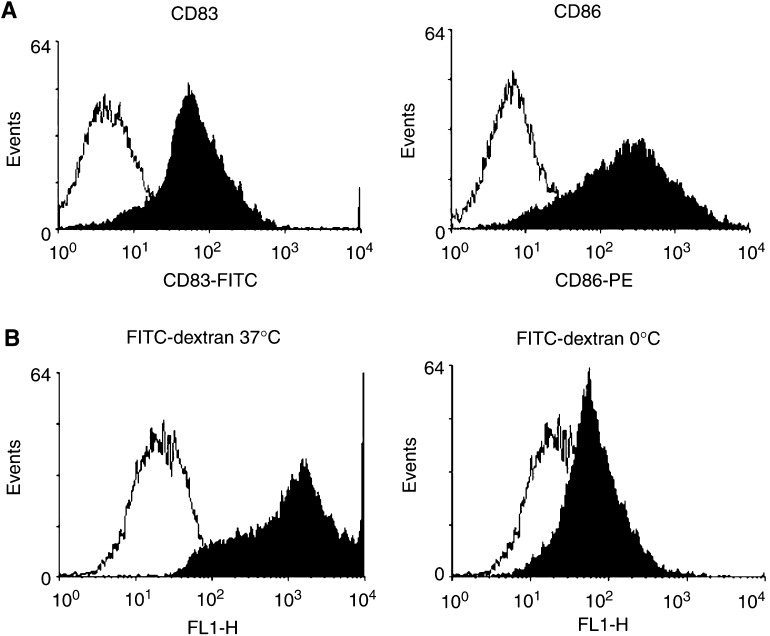
Flow cytometric plots showing the expression of DC markers CD83 and CD86 (**A**) and uptake of FITC-dextran (**B**) by unstimulated C3H/HeSn DCs.

Murine bone marrow progenitor cells were cultured in GM-CSF and IL-4 for 7 days to provide a population of DCs. A population of adherent, granular cells with surface dendrites was visible on examination by light microscopy (not shown). Flow cytometry was employed to examine the expression of the DC markers CD83 and CD86 (B7-2). Representative flow cytometric histograms are shown (Figure 1A

). The expression of these markers was similar in both C3H/HeSn and C3H/HeJ DC cultures.

Dendritic cell cultures were also examined for their ability to phagocytose antigen. It has previously been reported that immature DC phagocytosis can be examined by the *in vitro* uptake of dextran molecules. Representative flow cytometric histograms show fluorescence of DCs after incubation with FITC-dextran at 37°C (Figure 1B). At 0°C, very limited uptake of FITC-dextran and fluorescence was seen (*P*<0.0001). Uptake of antigen by C3H/HeSn and C3H/HeJ DCs did not differ significantly (*P*=0.1769). Together, the phenotypic marker expression and endocytosis assays support the hypothesis that a semimature population of DC is present using these *in vitro* culture conditions.

### BCG induces phenotypic maturation of DCs

**Figure 2:**
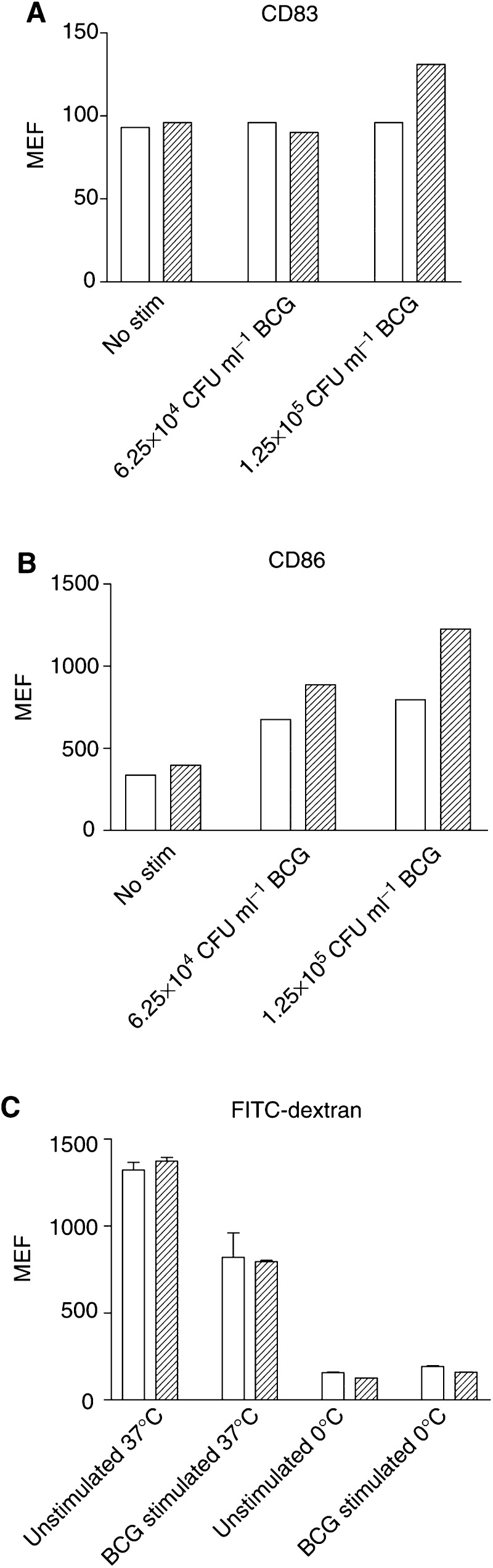
Expression of CD83 (**A**), CD86 (**B**) and uptake of FITC-dextran (**C**) by C3H/HeSn and C3H/HeJ DCs following stimulation with BCG. Dendritic cells were harvested from C3H/HeSn mice (clear bars) and C3H/HeJ mice (hatched bars) and cultured for 7 days in GM-CSF and IL-4 followed by stimulation with 6.25 × 10^4^ and 1.25 × 10^5^ CFU ml^−1^ BCG for 72 h. Expression of markers and FITC-dextran uptake were analysed by flow cytometry (MEF: median fluorescence; CFU: colony forming units).

Dendritic cells were examined for phenotypic maturation following stimulation by activating stimuli. This was determined by measuring the upregulation of CD83/86 marker expression and the downregulation of phagocytosis. Bacillus Calmette–Guerin stimulation of C3H/HeSn and C3H/HeJ cultures showed no difference on the maturation status of DC by examination of CD83 expression (Figure 2A

), but did result in a dose-dependent increase in the expression of CD86 in DCs from both mouse strains (Figure 2B).

Phenotypic maturation of DCs also results in the downregulation of antigen phagocytosis. A reduction in phagocytosis of FITC-dextran was observed in DCs from both mouse strains following 72 h BCG stimulation compared to nonstimulated control DC cultures (C3H/HeSn; *P*=0.0133, C3H/HeJ; *P*<0.0001) (Figure 2C). C3H/HeSn and C3H/HeJ did not differ in their activation and downmodulation of phagocytosis after BCG stimulation (*P*=0.4334).

### Antigen presentation by BCG stimulated DCs

**Figure 3:**
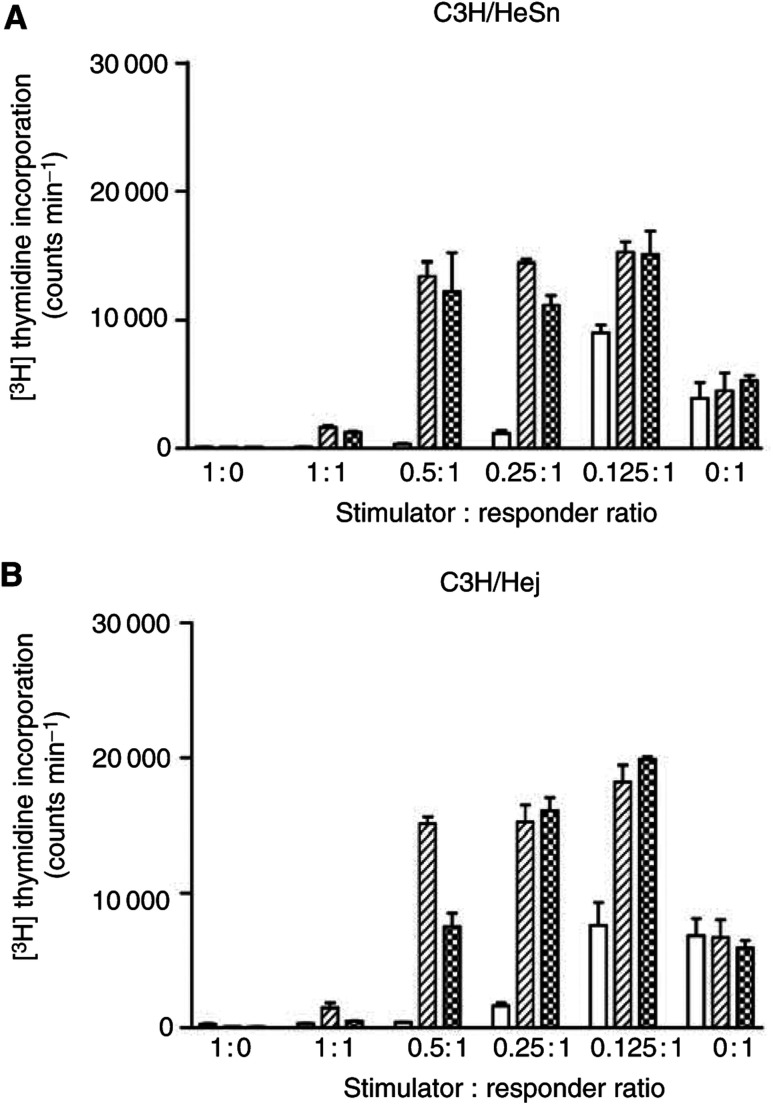
Antigen presentation by C3H/HeSn (**A**) and C3H/HeJ (**B**) DCs in the presence and absence of BCG. Functional antigen presentation was assessed by assessment of murine CD1 T-cell proliferation in an allogenic MLR. Dendritic cells were cultured for 7 days in the presence of GMCSF and IL-4, followed by culture for 72 h in either the absence of activating stimuli (clear bars), 6.25 × 10^4^ CFU ml^−1^ BCG (hatched bars) or 1.25 × 10^5^ CFU ml^−1^ BCG (checked bars). Proliferation of T cells was measured by the incorporation of [3H]thymidine.

Mixed lymphocyte reaction assays were carried out to assess the functional antigen-presentation state of DC cultures to induce the proliferation of resting allogeneic T cells in coculture with BCG stimulated C3H/HeSn and C3H/HeJ DCs (Figure 3

). Bacillus Calmette–Guerin-stimulated C3H/HeSn and C3H/HeJ DCs have an increased capacity to induce significant T-cell proliferation at stimulator: responder ratios of 0.5 : 1 and below, compared to nonstimulated DCs (0.5 : 1, *P*=0.0078 and 0.0010, respectively, 0.125 : 1, *P*=0.0175 and 0.0010, respectively). This induction of proliferation did not differ significantly between C3H/HeSn and C3H/HeJ DCs (*P*=0.1010). No induction of proliferation was evident at 1 : 1 stimulator : responder ratio. No proliferation was observed in control cultures of DC alone, confirming that 25 Gy *γ*-irradiation is sufficient to prevent DC proliferation.

### Activation of DCs by BCG produces both Th1- and Th2-polarising cytokines

**Figure 4:**
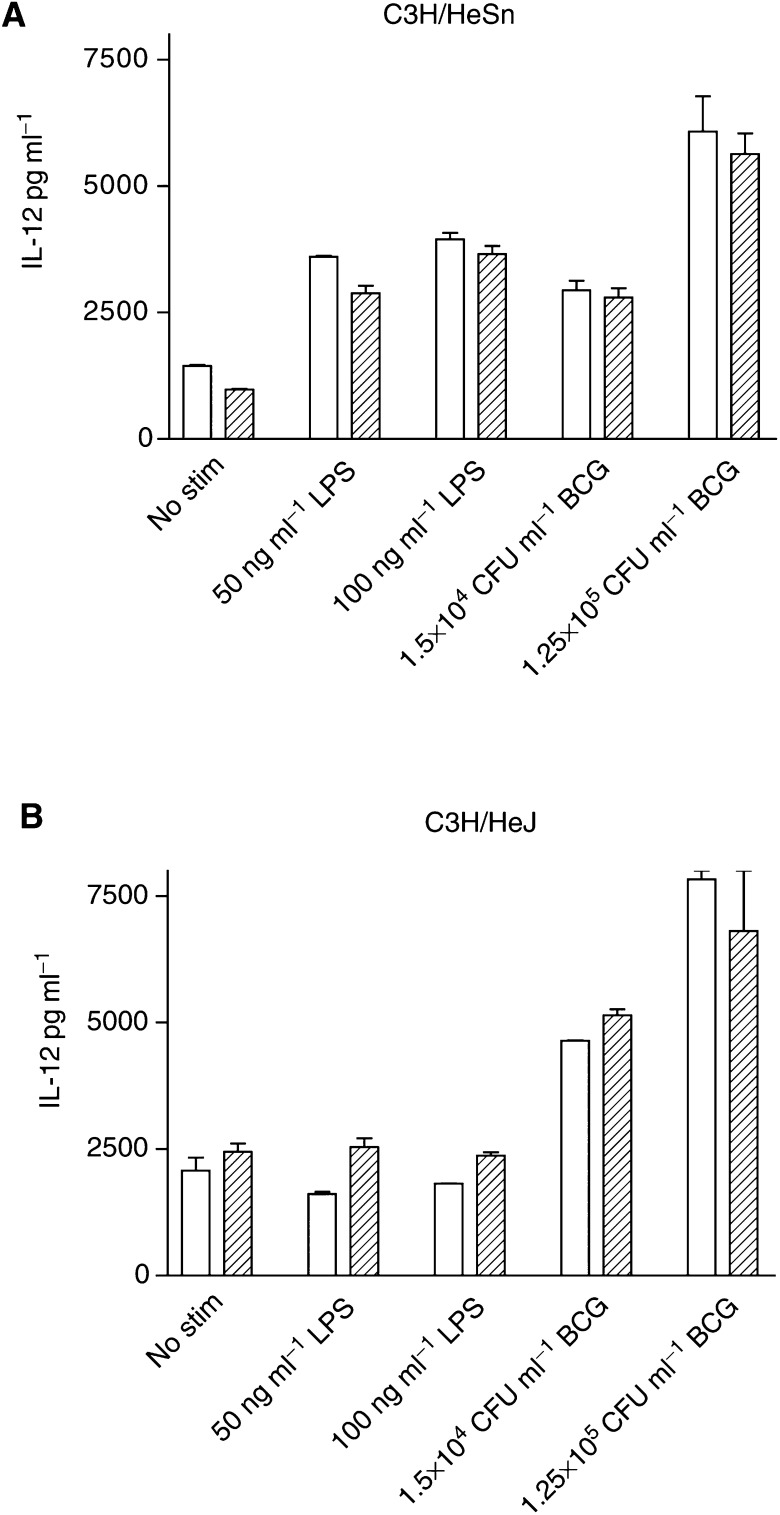
Production of IL-12 by C3H/HeSn (**A**) and C3H/HeJ (**B**) DCs following stimulation with LPS and BCG. Dendritic cells were cultured for 7 days in the presence of GM-CSF only (clear bars) or GM-CSF and IL-4 (hatched bars), followed by 72 h stimulation with either LPS or BCG. IL-12 concentration was determined by ELISA.

Secretion of the Th1-polarising cytokine IL-12 and the Th2-polarising cytokine IL-10 from C3H DCs stimulated with BCG or LPS was studied. IL-12 could be detected in culture supernatants 24 h after stimulation (data not shown) and after this, no further increase in cytokine production was observed. IL-12 production was enhanced by C3H/HeSn DCs in response to both LPS and BCG compared to nonstimulated control cultures (*P*=0.0001 and 0.0006, respectively) (Figure 4A

). Stimulation of IL-12 by LPS-treated C3H/HeSn DCs was significantly greater when IL-4 was omitted from the culture medium (*P*=0.004), whereas stimulation of IL-12 production by BCG in DCs from this strain was unaffected by the presence of IL-4 (*P*=0.301). The TLR4 mutant C3H/HeJ DCs also showed enhanced IL-12 production in response to BCG (*P*<0.0001) but, as expected, no enhancement of IL-12 production was observed in response to the TLR4 ligand LPS (*P*=0.356) (Figure 4B).

**Figure 5:**
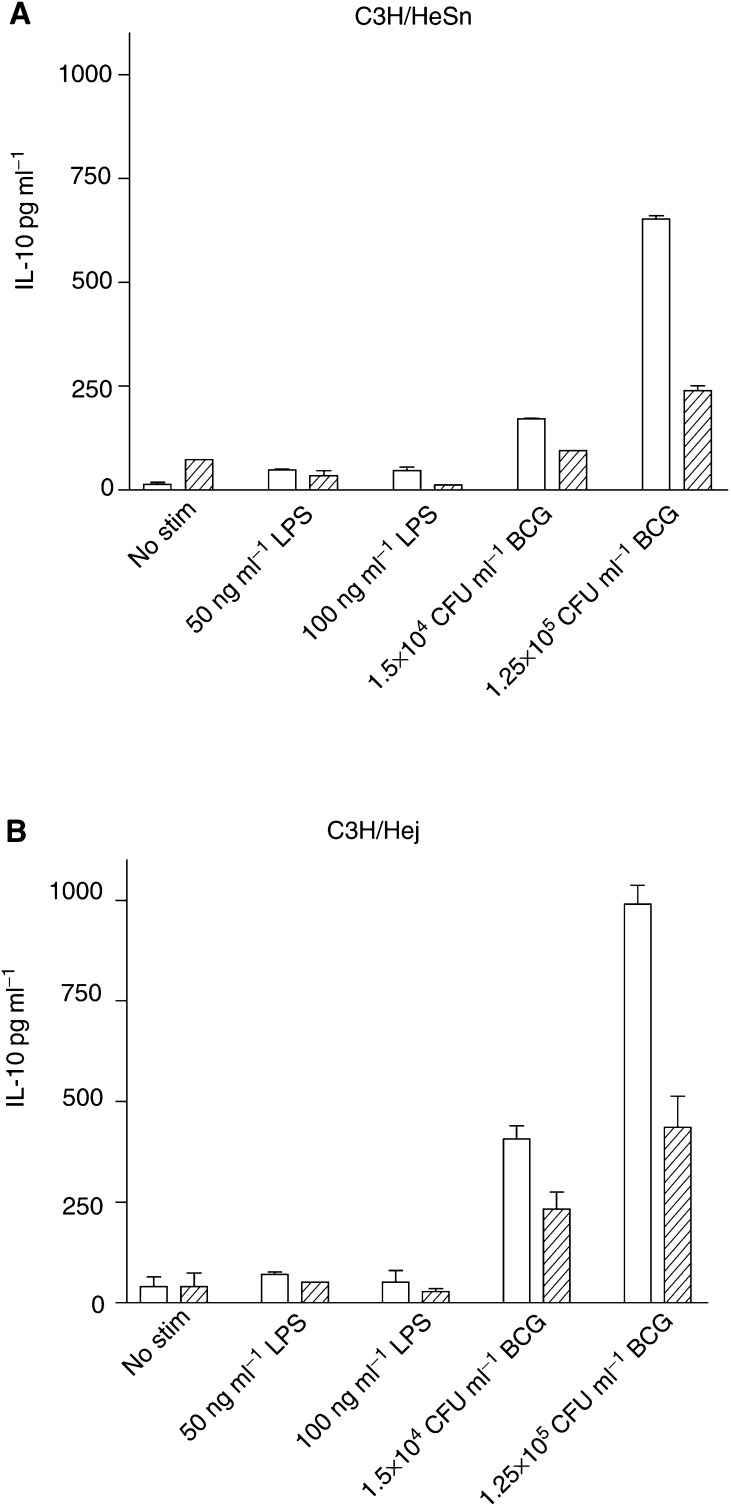
Production of IL-10 by C3H/HeSn (**A**) and C3H/HeJ (**B**) DCs following stimulation with LPS and BCG. Dendritic cells were cultured for 7 days in the presence of GM-CSF only (clear bars) or GM-CSF and IL-4 (hatched bars), followed by 72 h stimulation with either LPS or BCG. IL-10 concentration was determined by ELISA.

In DCs from both mouse strains, IL-10 production was enhanced by BCG (*P*<0.0001), but not by LPS (*P*=0.3829) (Figure 5

). IL-10 production was also dependent on the cytokine conditions of the DC cultures. The induction of IL-10 was significantly lower when DCs were cultured in the presence of both GM-CSF and IL-4 conditions compared to GM-CSF alone (*P*<0.0001).

### Activation of DCs by CpG-oligonucleotides produces a polarised Th1 cytokine response

**Figure 6:**
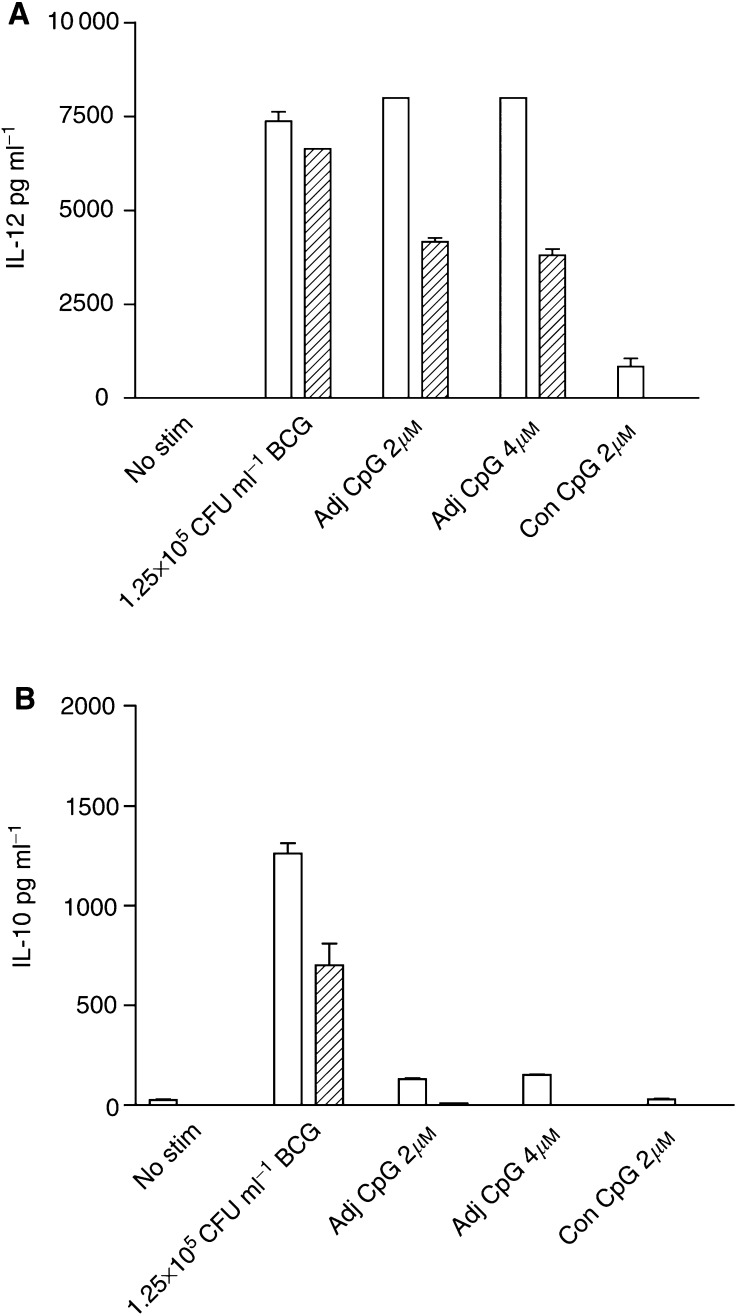
Production of IL-12 (**A**) and IL-10 (**B**) by C3H/HeSn following stimulation with CpG-oligonucleotides. Dendritic cells were cultured for 7 days in the presence of GM-CSF only (clear bars) or GM-CSF and IL-4 (hatched bars), followed by 72 h stimulation with either 1.25 × 10^5^ CFU ml^−1^ BCG, 2 or 4 *μ*M adjuvant CpG or 2 *μ*m control oligonucleotide. Cytokine concentration was determined by ELISA.

The activation of DCs by phosphorothioate-stabilised, adjuvant CpG-containing oligonucleotide was also assessed and measured by cytokine production. Following stimulation of C3H/HeSn DCs with adjuvant CpG-oligonucleotides, the production of IL-12 was comparable to that achieved following BCG stimulation and was highly significant compared to control cultures (*P*<0.0001) (Figure 6A

). The amount of secreted IL-12 in response to both BCG or adjuvant CpG did not differ between the two C3H mouse strains (data not shown). Stimulation of DCs with oligonucleotides lacking CpG-motifs failed to induce IL-12 production compared to CpG-containing oligonucleotides (*P*<0.0001). As with BCG stimulation, IL-12 production in response to adjuvant CpG-oligonucleotides was dependent on the *in vitro* cytokine conditions, with a significantly lower induction of IL-12 in GM-CSF and IL-4 conditions (*P*<0.0001).

In contrast to stimulation with BCG, CpG-oligonucleotide stimulation did not result in IL-10 production in C3H/HeSn DCs (Figure 6B; *P*<0.0001). This apparently polarised Th1 response was even more striking when the DCs were cultured in GM-CSF and IL-4 rather than GM-CSF alone. A summary of cytokine production by *in vitro* DC cultures from both mouse strains following stimulation with different TLR ligands is given in Table 1

**Table 1:** Cytokine production by cultured DCs from TLR4 mutant and wild-type C3H mice following stimulation by LPS, BCG and CpG-oligonucleotides

	**C3H/HeN: TLR4 wild type/LPS responsive**		**C3H/HeJ: TLR4 mutant/LPS resistant**	
	**IL-12**	**IL-10**	**IL-12**	**IL-10**
BCG	+	+	+	+
LPS	+	−	−	−
CpG	+	−	+	−

DC=dendritic cells; TLR=toll-like receptor; LPS=lipopolysaccharide; BCC=bacillus Calmette–Guerin; IL=interleukin.

.

## DISCUSSION

We have developed a reliable and reproducible method for the culture and activation of DC from murine bone marrow progenitors. These cultures display the highly phagocytic phenotype characteristic of immature DCs but also express CD83 and CD86, which are markers of DC maturation. This supports previous findings that DCs cultured *ex vivo* are of a semimature phenotype (Son *et al*, 2002). Semimature DC phenotypes are analogous to steady-state migratory veiled DCs within lymphatics and are thought to tolerise lymph node T cells against tissue-derived self-antigens or apoptotic cells (Inaba *et al*, 1998; Lutz and Schuler, 2002). Such DCs are nonimmunogenic and are distinguished as mature by surface marker expression (MHCII^high^ and costimulation^high^), but have no elevated release of proinflammatory cytokines. They require decisive immunogenic maturation signals for full maturation and the release of proinflammatory cytokines. The activation of DCs also results in the downmodulation of endocytosis and antigen processing, stablisation of MHC peptide complexes and upregulation of molecules related to antigen presentation and T-cell costimulation (Fearon and Locksley, 1996).

Several TLR ligands including BCG, CpG and LPS are potent stimuli for DC activation and induce both a mature phenotype and cytokine production. In this study, we have shown that BCG stimulation of DCs induces maturation from a semimature to a fully mature phenotype, which is evident by downregulation of phagocytosis and upregulation of the maturation marker CD83 and costimulatory molecule CD86. We have also shown that phenotypic changes associated with the maturation of DCs are similar in both C3H/HeSn (TLR4 wild type) and C3H/HeJ (TLR4 mutant) DCs following activation with BCG. Genetic analysis of the endotoxin hyposensitive mouse C3H/HeJ identified TLR4 as the primary receptor for LPS (Qureshi *et al*, 1999). This demonstrates that BCG activation of antigen presentation by DCs is not dependent on TLR4 engagement.

Our investigations have also shown that BCG stimulation of murine DC cultures results in the production of a proinflammatory cytokine milieu, but which is not polarised towards a Th1-inducing profile. This is evident from the production of both IL-12 (Th1) and IL-10 (Th2) cytokines. In clinical practice, a burst of urinary Th1 cytokines IFNγ, IL-12 and IL-2 has been observed after BCG therapy and is a common feature in BCG responders, whereas higher levels of the Th2 cytokines IL-10 and/or IL-6 appear to be associated with BCG failure (Esuvaranathan *et al*, 1995; de Reijke *et al*, 1996). Patients who failed BCG immunotherapy also showed higher antibody responses to BCG heat shock proteins in their sera (Zlotta *et al*, 1997). This nonpolarised activation of immune cells may represent a mechanism for BCG treatment failure in bladder cancer patients. Thus, it is proposed that efforts to polarise the immune response by increasing the production of Th1-promoting IL-12, and diminishing IL-10 will have a therapeutic value. Indeed, IL-10 deletion studies performed either by antibody inhibition or using gene knockout animals resulted in enhanced DTH response and antitumour activity (Nadler *et al*, 2003), while IL-10 knockout mice also showed prolonged survival and response in a syngeneic model of orthotopic bladder cancer (Riemensberger *et al*, 2002). This response was shown to coincide with an increased influx of T and NK cells into the bladder walls of these mice.

Another mechanism for promoting a Th1 immune response involves the therapeutic use of cytokine combinations to increase the production of IFN*γ*. O'Donnell *et al* (1999) reported a striking increase in IFNγ production in cell culture following the addition of small amounts of recombinant IL-12. This synergistic effect of IFNγ production could also be produced by intravesical administration of BCG with IL-12, but was downregulated by IL-10 (O'Donnell *et al*, 1999). The ability of exogenous IL-12 to shift this balance strongly towards Th1 provides the immunological basis for combination with BCG for successful bladder cancer therapy.

It has been reported that nonmethylated palindromic DNA containing CpG-ODN can activate an innate immune response by activation of NK cells, DC and B cells in an antigen-independent manner (Weiner *et al*, 1997). Recognition of these microbial sequences is mediated by a member of the TLR family, namely TLR9. CpG-oligonucleotides have been shown to act as adjuvants to induce the expression of Th1-type cytokines in mouse models (Chu *et al*, 1997), and lead to the production of IL-12, IL-18 and IFN*γ* by peripheral blood mononuclear cells in humans (Bohle *et al*, 1999). CpG-ODN have also been used to produce cancer-antigen-presenting DCs for use in immunotherapy (Brunner *et al*, 2000). Further studies have shown that peritumoral treatment resulted in complete rejection or strong inhibition of a variety of established mouse tumours, including B16 melanoma and 3LL lung carcinoma, whereas systemic administration only had partial effects (Kawarada *et al*, 2001). Heckelsmiller *et al* (2002) reported the combined use of CpG and antigen-pulsed DCs to cure large murine tumours that were resistant to chemotherapy.

**Figure 7:**
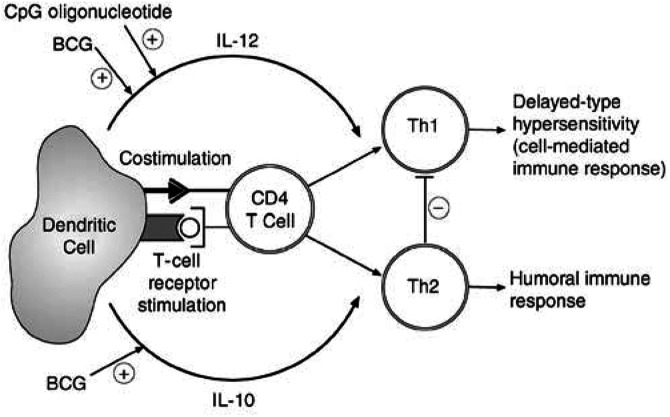
CpG-oligonucleotides activate DCs and preferentially elicit IL-12 production, which leads to the polarisation of a Th1, tissue-destructive and cancer-clearing immune phenotype. This is in contrast to BCG, which induces a poorly focused T-cell response containing both Th1 and Th2 immune function.

Phosphorothioate-stabilised CpG-oligonucleotide sequences activate and preferentially elicit Th1 cytokine production by cultured mouse DCs (Jones *et al*, 2002). In our studies, we have shown that CpG-ODN stimulation of murine DC cultures results in the production of the Th1-polarising cytokine IL-12 at levels comparable to those produced by BCG. However, in contrast to BCG, CpG-oligonucleotides did not result in concurrent production of the Th2 cytokine IL-10 (Figure 7

). An inverted control sequence motif oligonucleotide failed to stimulate DC cultures to produce a cytokine response, showing that this effect was sequence specific. As expected, cytokine production was independent of TLR4 status as shown by IL-12 production in both C3H/HeSn and C3H/HeJ DCs. However, although CpG-ODN did induce a polarised Th1 cytokine response, this was dependent on the *in vitro* culture cytokine conditions employed. We observed that IL-12 secretion from DCs was only evident when DCs were cultured in GM-CSF alone. The presence of the Th2 cytokine IL-4 appeared to inhibit IL-12 production in response to CpG-ODN stimulation. This suggests that the Th1-polarising stimulation of DCs by CpG will be dependent on the local cytokine environment of the DC activation.

The finding that CpG-oligonucleotides can induce IL-12 production in DCs without concurrent production of IL-10 supports our hypothesis that DCs can be polarised to a Th1 immune response and that this can be manipulated to improve the efficiency of bladder cancer immunotherapy. The potential of CpG-oligonucleotides as a single agent or combination therapy with sparing doses of BCG is currently being evaluated in an orthotopic mouse model of bladder cancer.
